# Sternal osteomyelitis and infective endocarditis after old trivial chest trauma in untreated diabetes mellitus: A case report

**DOI:** 10.1002/jgf2.347

**Published:** 2020-06-07

**Authors:** Ibuki Kurihara, Masahiro Kashiura, Takashi Moriya, Hitoshi Sugawara

**Affiliations:** ^1^ Division of General Medicine Department of Comprehensive Medicine 1 Saitama Medical Center Jichi Medical University Saitama Japan; ^2^ Department of Emergency and Critical Care Medicine Saitama Medical Center Jichi Medical University Saitama Japan

**Keywords:** chronic sternal osteomyelitis, diabetes mellitus, heart valve replacement, infective endocarditis, *Staphylococcus aureus*

## Abstract

An 82‐year‐old man with untreated diabetes mellitus (DM) had anterior chest wall swelling and ulcers 2 years following blunt chest trauma. Contrast‐enhanced computed tomography revealed sternal fracture with osteolytic change and subcutaneous abscess. Blood and sternal cultures were positive for methicillin‐susceptible *Staphylococcus aureus* (MSSA). Transesophageal echocardiogram showed vegetation on the right coronary cusp and moderate aortic regurgitation. The patient received a diagnosis of infective endocarditis associated with chronic sternal osteomyelitis complicated by subcutaneous abscess because of MSSA. This case report showed that trivial trauma in patients with uncontrolled DM can cause chronic sternal osteomyelitis resulting in infective endocarditis.

## Graphical abstract

Trivial trauma of the sternum in patients with uncontrolled DM may cause late‐onset sternal osteomyelitis resulting in infective endocarditis. Physicians should consider a history of trivial trauma in the sternum in patients with uncontrolled DM because it can lead to late‐onset sternal osteomyelitis, which subsequently causes infective endocarditis.

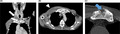

## 1 INTRODUCTION

Primary sternal osteomyelitis is a rare condition that occurs in only 0.3% of individuals with osteomyelitis.^1^ Few studies have reported chronic primary sternal osteomyelitis following trivial trauma.^1^, ^2^ The occurrence of infective endocarditis with primary sternal osteomyelitis is uncommon.^2^, ^3^ Here, we describe the case of an 82‐year‐old man who developed infective endocarditis associated with sternal osteomyelitis 2 years after trivial chest trauma.

## 2 CASE REPORT

An 82‐year‐old Japanese man received a diagnosis of diabetes mellitus (DM) based on a fasting blood glucose level of 145 mg/dL and HbA1c of 6.7% 13 years back and was not treated for the same. Two years before admission, he sustained a bruise to his anterior sternum from a car accident; although he did not consult a doctor at the time, his condition spontaneously improved. Three months before admission, he experienced anterior chest wall swelling and ulcers on the sternum with exudation and consulted a dermatologist. The sternal trauma fluid revealed the presence of methicillin‐susceptible *Staphylococcus aureus* (MSSA). The Infectious Diseases Society of America guideline has recommended a first‐generation cephalosporin or antistaphylococcal penicillin for skin and soft tissue MSSA infections.^4^ Unfortunately, the patient was administered 500 mg of oral levofloxacin daily for 1 week. The swelling subsequently worsened; 9 days before admission, he was unable to lift his right shoulder. The patient was subsequently admitted to our hospital.

**Figure 1:**
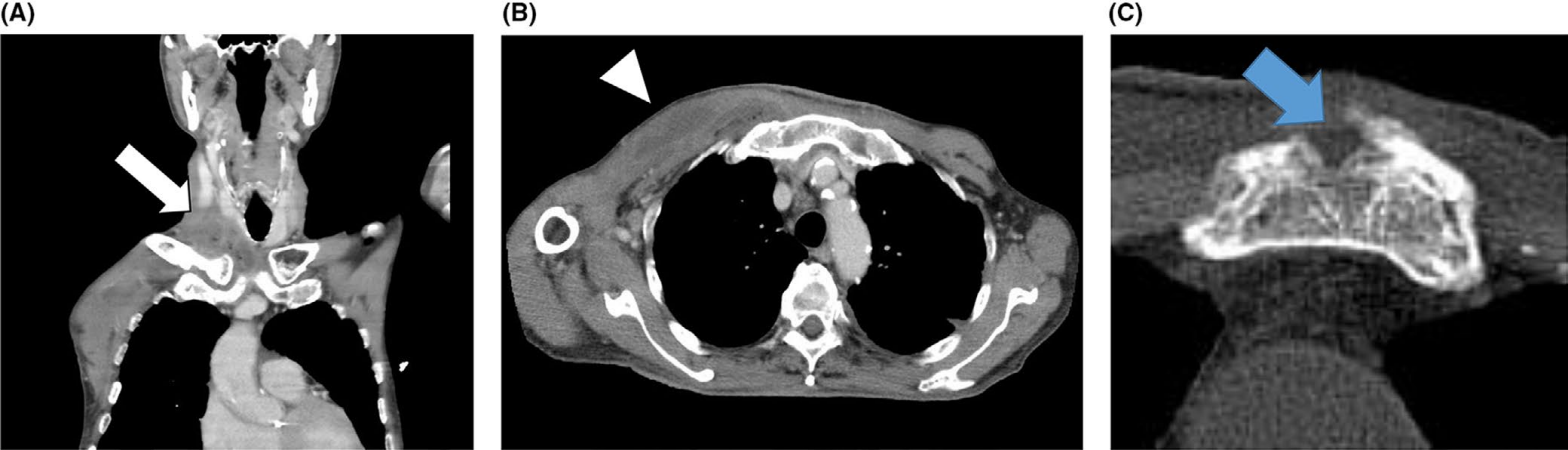
A, Contrast‐enhanced CT scan upon admission showing the coronal section of a neck abscess (white arrow). B, Transverse section of a chest wall abscess (white arrowhead). C, Old sternal fracture with osteolytic change (blue arrow)

Physical examination showed a temperature of 36.7°C and a 1.5‐cm ulcer with sternal exposure and a warm, swollen, and erythematous bulge spreading from the ulcer to the right shoulder and neck. Further examination identified Levine 3/6 systolic ejection murmur at the right sternal border in the second intercostal space and no Osler node or Janeway lesion. Laboratory test results included a white blood cell count of 20.39 × 10^3^/μL (neutrophils, 96%), blood urea nitrogen/serum creatinine level of 31/0.98 mg/dL, C‐reactive protein level of 30.1 mg/dL, blood glucose level of 247 mg/dL, and hemoglobin A1c of 9.7%. Contrast‐enhanced computed tomography (CT) revealed sternal fracture with osteolytic change and chest abscess spreading from the sternal fracture to the right sternoclavicular joint and neck (Figure 1).

**Figure 2:**
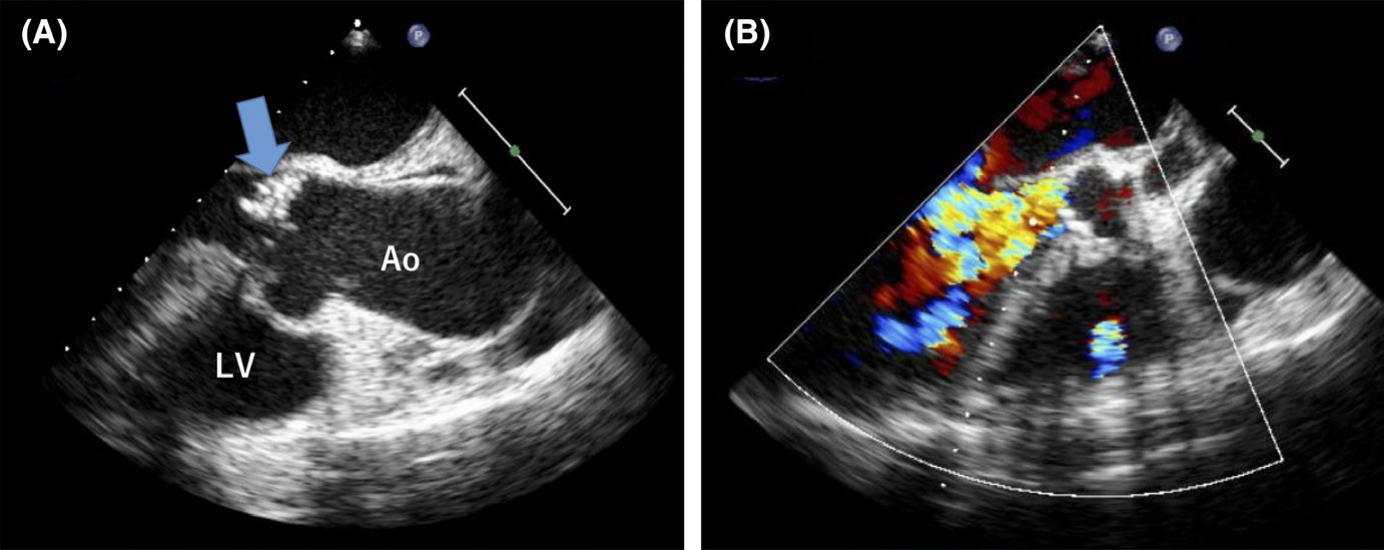
A, Transesophageal echocardiography on day 36 showing a 1.5‐cm motile vegetation (blue arrow) on the noncoronary cusp of the aortic valve. B, Severe atrial regurgitation

Abscess subcutaneous drainage and extensive sequestrectomy of the sternum were performed, and 1 g of intravenous vancomycin every 12 hours and 2 g of ceftriaxone every 24 hous were administered on day 1. On day 2, transesophageal echocardiogram showed vegetation on the right coronary cusp and moderate aortic regurgitation (AR). All five sets of blood cultures tested positive for MSSA. These findings met two major and one minor Duke criteria for definitive diagnosis of endocarditis.^5^ The patient received a diagnosis of infective endocarditis‐associated sternal osteomyelitis caused by MSSA. We consulted with an infectious disease specialist, and the patient was intravenously treated with 2 g of cefazolin every 8 hours on day 4. The patient underwent additional abscess drainage and total sequestrectomy in the sternum on days 7 and 15, respectively. Blood culture was negative on day 8; however, AR became severe, causing hemodynamic instability and respiratory failure (Figure 2). He underwent aortic valve replacement with a bioprosthetic valve via right thoracotomy on hospital day 38. Macroscopic findings revealed exenterated aortic valve and perforated noncoronary and right coronary cusps. On day 68, sternal closure using an advanced flap grafting was performed. Contrast‐enhanced CT revealed abscess disappearance, and we completed the antibiotic therapy on day 104. The patient received 36‐day cefazolin since the sternal closure was performed on day 68. It is critical but often missed by cardiothoracic surgeons to perform the culture of the infected heart valve because the American Heart Association (AHA) guidelines indicate additional antibiotic therapy for 14 days after valve replacement.^6^ Unfortunately, we did not perform a culture of the infected heart valve. We administered additional antibiotics therapy for 66 days, over 14 days, after valve replacement according to the AHA guidelines.^6^ The patient was transferred to a rehabilitation hospital on day 116.

## 3 DISCUSSION

Trivial trauma of the sternum in patients with uncontrolled DM may cause chronic sternal osteomyelitis resulting in infective endocarditis. The present case highlights two important facts. First, minor trauma can cause chronic sternal osteomyelitis. Second, sternal osteomyelitis can lead to bloodstream infection and infective endocarditis in patients with uncontrolled DM.

First, although *S aureus* can migrate from any type of skin breakdown, the occurrence of chronic sternal osteomyelitis after trivial trauma is extremely rare. Only two such cases have been reported, and the pathogen in both cases was *S aureus* (Table S1).^1^, ^2^ Only the patient in this report had sternal osteomyelitis associated with infective endocarditis. The duration of antibiotic treatment was 45‐116 days, and none of the patients were treated with antimicrobial chronic suppression. Trauma causes vascular insufficiency and bone fragility and necrotic bone fragments, thereby forming an ideal environment for bacterial growth,^7^ which could have caused chronic sternal osteomyelitis in our patient.

Second, sternal osteomyelitis can lead to bloodstream infection and infective endocarditis in patients with uncontrolled DM. The present patient's long‐term history of DM probably contributed to promoting *S aureus* infection. Two cases of sternal osteomyelitis causing bacteremia and knee abscesses have been reported; however, sternal osteomyelitis rarely causes disseminated lesions.^8^, ^9^ Smit et al^10^ reported an association between DM and community‐acquired *S aureus* bacteremia, particularly in patients with long‐term DM, poor glycemic control, or complications of diabetes. In this case, the patient's immunosuppressive status of untreated DM may have contributed to the development of *S aureus* bacteremia, which is a substantial predisposing factor for infective endocarditis.

In conclusion, physicians should consider a history of trivial trauma in the sternum in patients with uncontrolled DM because it can lead to chronic sternal osteomyelitis, which subsequently causes infective endocarditis.

## CONFLICT OF INTEREST

Authors declare no conflict of interests for this article.

## Supporting information

Table S1
