# Supplementary material for: Sternal osteomyelitis and infective endocarditis after old trivial chest trauma in untreated diabetes mellitus: A case report
Source: J Gen Fam Med. 2020 Jun 7;21(5):188–90. doi: 10.1002/jgf2.347 (PMC7521787; doi:10.1002/jgf2.347)
Supplement: Supplementary file 1 — Table S1 [file JGF2-21-188-s001.docx]

Supplementary Table 1. Previously published case reports of late-onset sternal osteomyelitis caused by trivial blunt trauma

| Case  number | Sex | Age (years) | Comorbidity | Mechanism of trauma | Duration from trauma to onset of osteomyelitis | | Distal seeding | Pathogens | Antibiotics |
| --- | --- | --- | --- | --- | --- | --- | --- | --- | --- |
| 1 [2] | Female | 36 | (−) | Bruise | 3 years | (−) | | *S. aureus* | Vancomycin,  Ciprofloxacin  for 45 days |
| 2 [3] | Male | 72 | DM | Impalement | >10 years | (−) | | *S. aureus* | Cefazoline  for 90 days |
| 3 [current patient] | Male | 82 | DM | Bruise | 2 years | Infective endocarditis | | *S. aureus* | Cefazoline  for 116 days |

DM: diabetes mellitus, *S. aureus*: *Staphylococcus aureus*
